# Supplementary material for: Effects of massive transfusion (10-20 litres) versus ultramassive transfusion (≥20 litres) on mortality in adult liver transplant recipients: A propensity-score matched study
Source: PLoS One. 2026 May 21;21(5):e0349795. doi: 10.1371/journal.pone.0349795 (PMC13193539; doi:10.1371/journal.pone.0349795)
Supplement: S1 Fig — (PDF) [file pone.0349795.s001.pdf]

Supplementary Figure 1. Unmatched analysis: Love plots of covariate balance across multiple propensity score matching strategies.

Standardised mean differences (SMDs) for all baseline covariates before and after matching are displayed for each propensity score matching configuration evaluated, comparing ultramassive transfusion (≥20 L of intraoperative fluids) with massive transfusion (10-20 L). The vertical dashed line at SMD = 0.1 indicates the threshold for adequate balance.

Optimal 1:1

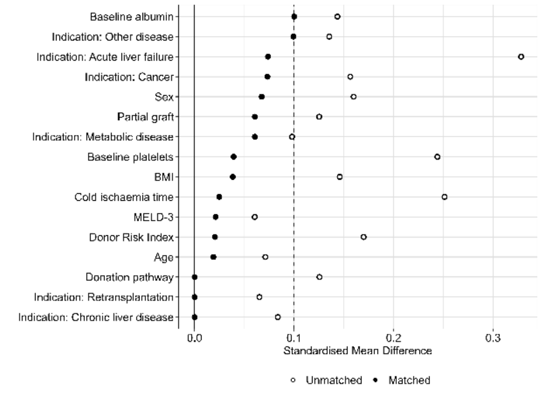

Optimal 2:1

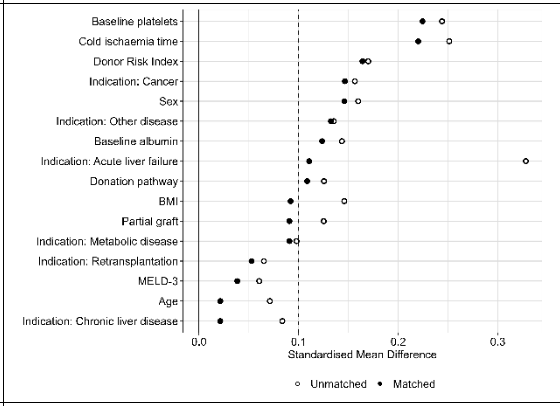

NNM 1:1

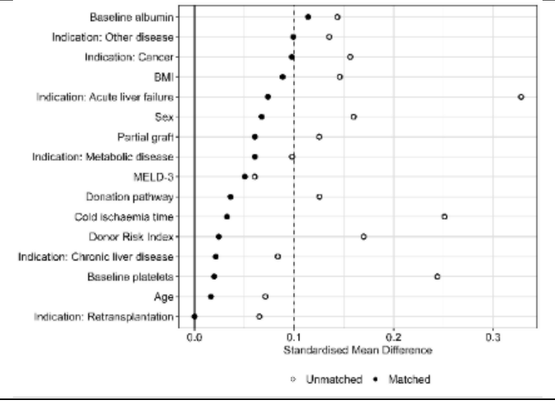

NNM 2:1

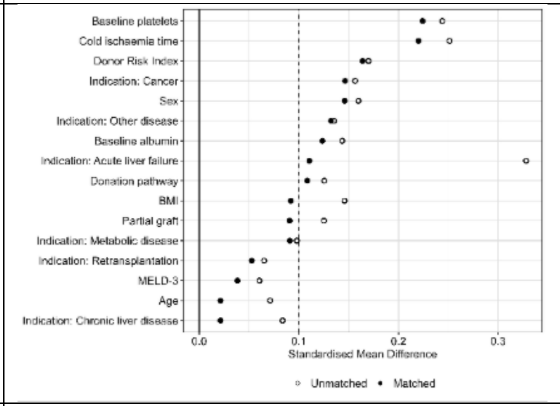

NNM 1:1 caliper 0.1

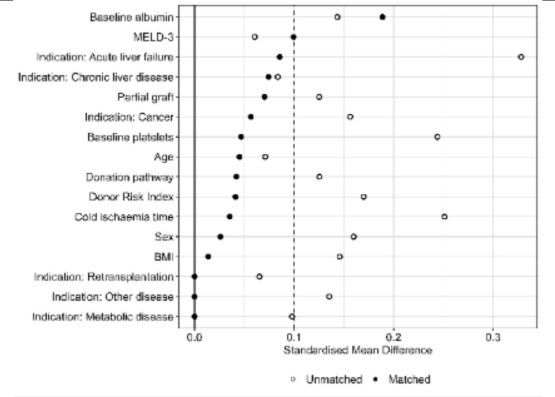

NNM 1:1 caliper 0.2

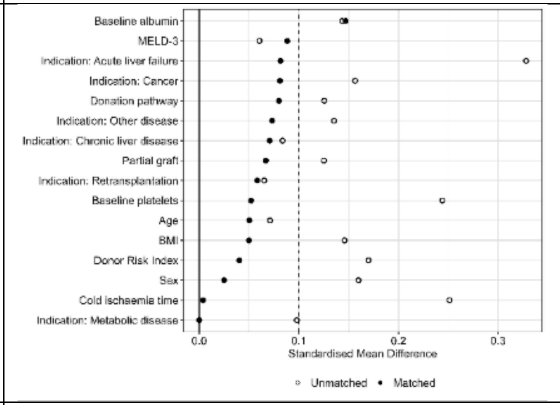

NNM 2:1 caliper 0.1

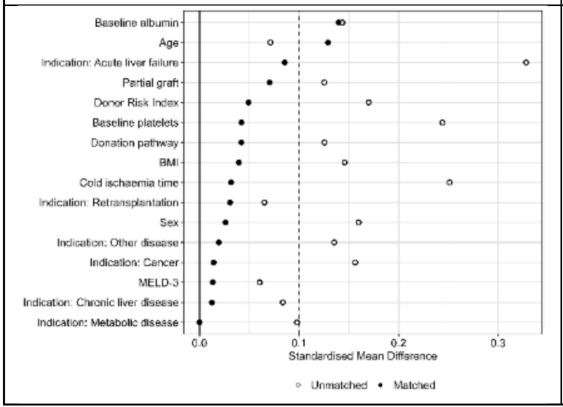

NNM 2:1 caliper 0.2

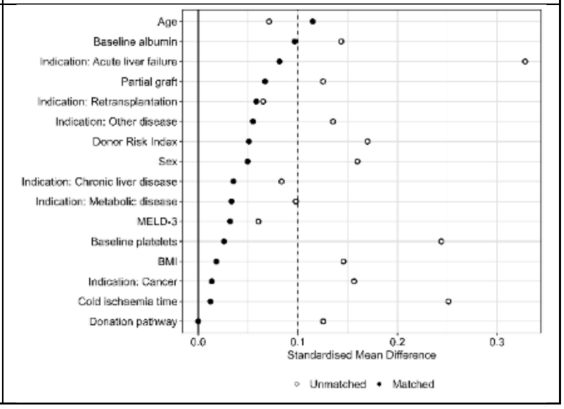

Abbreviations: NNM; nearest neighbour matching
