# Supplementary material for: Effects of massive transfusion (10-20 litres) versus ultramassive transfusion (≥20 litres) on mortality in adult liver transplant recipients: A propensity-score matched study
Source: PLoS One. 2026 May 21;21(5):e0349795. doi: 10.1371/journal.pone.0349795 (PMC13193539; doi:10.1371/journal.pone.0349795)

Supplementary Figure 2. Primary analysis: Long-term survival comparisons in the propensity-matched cohort.

Kaplan-Meier curves displaying overall survival probability over the entire follow-up period in the propensity-matched cohort, comparing ultramassive transfusion (UMT; ≥20 L of intraoperative fluids) with massive transfusion (MT; 10–20 L of intraoperative fluids). Log-rank *p*-values are provided in each panel. (A) Overall patient survival: log-rank $p = 0.012$. (B) Overall graft survival: log-rank $p = 0.114$.

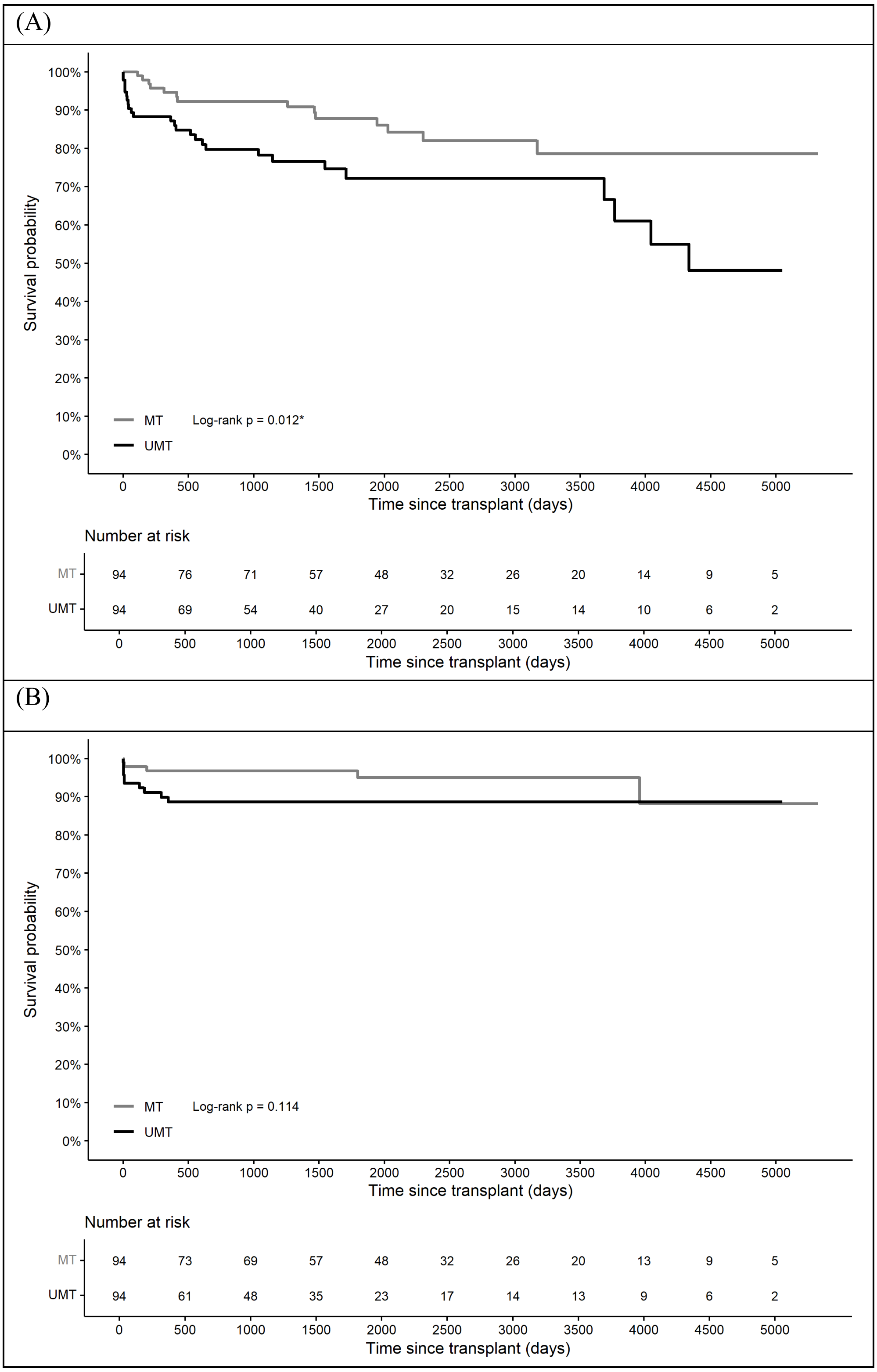

Supplement: S2 Fig — (PDF) [file pone.0349795.s002.pdf]
