# Supplementary material for: Effects of massive transfusion (10-20 litres) versus ultramassive transfusion (≥20 litres) on mortality in adult liver transplant recipients: A propensity-score matched study
Source: PLoS One. 2026 May 21;21(5):e0349795. doi: 10.1371/journal.pone.0349795 (PMC13193539; doi:10.1371/journal.pone.0349795)

Supplementary Figure 3. Unmatched analysis: Early, intermediate and long-term survival comparison in the unmatched cohort.

Kaplan-Meier curves displaying survival probability in the unmatched cohort, comparing ultramassive transfusion (UMT; ≥20 L of intraoperative fluids) with massive transfusion (MT; 10-20 L of intraoperative fluids). Log-rank *p*-values are provided in each panel. (A) 90-day patient survival: log-rank $p < 0.001$. (B) 3-year patient survival: log-rank $p < 0.001$. (C) Overall patient survival: log-rank $p < 0.001$. (D) 90-day graft survival: log-rank $p = 0.519$. (E) 3-year graft survival: log-rank $p = 0.073$. (F) Overall graft survival: log-rank $p = 0.149$.

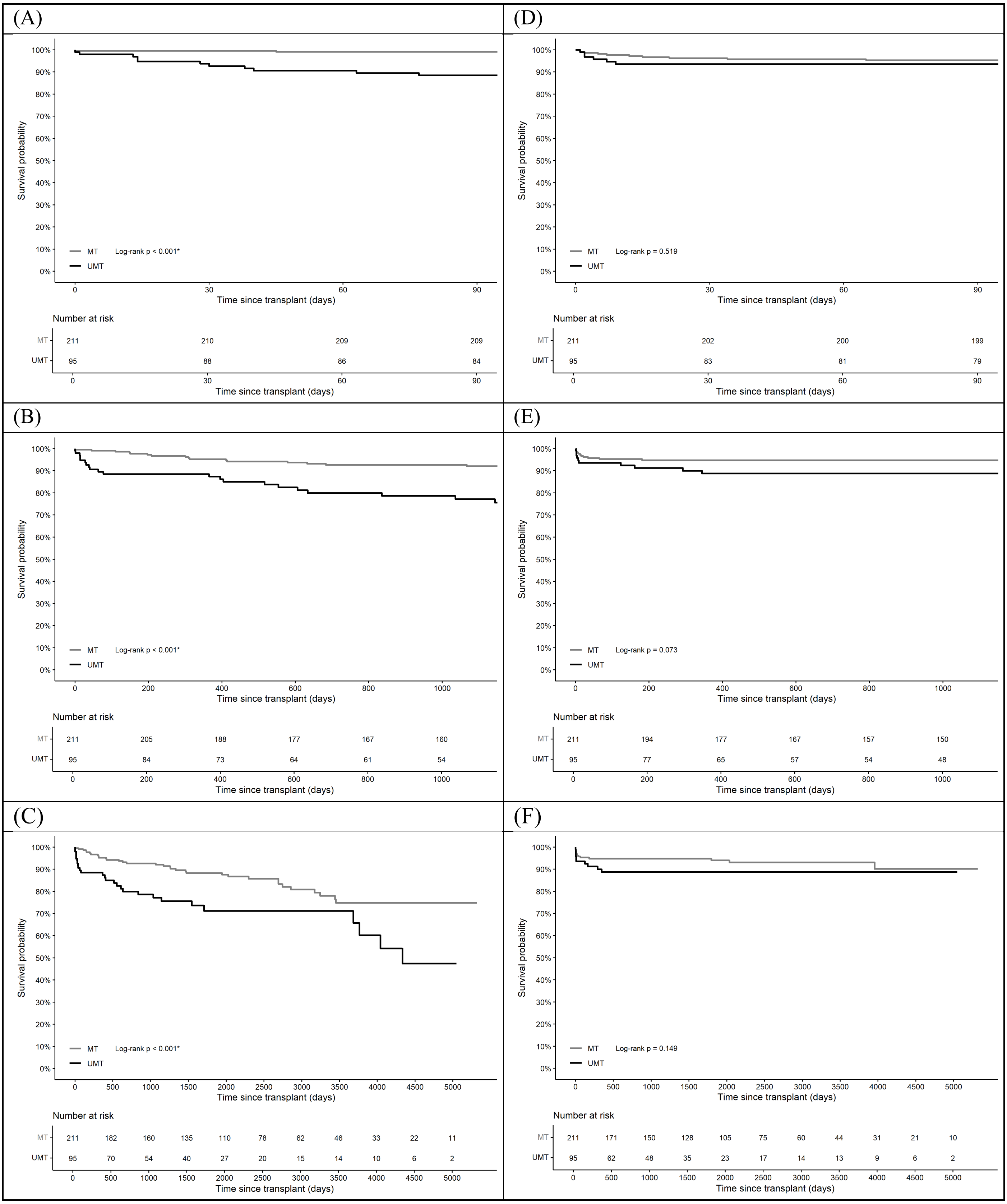

Supplement: S3 Fig — (PDF) [file pone.0349795.s003.pdf]
