# Supplementary material for: Effects of massive transfusion (10-20 litres) versus ultramassive transfusion (≥20 litres) on mortality in adult liver transplant recipients: A propensity-score matched study
Source: PLoS One. 2026 May 21;21(5):e0349795. doi: 10.1371/journal.pone.0349795 (PMC13193539; doi:10.1371/journal.pone.0349795)
Supplement: S5 Fig — (PDF) [file pone.0349795.s005.pdf]

Supplementary Figure 5. Sensitivity analysis II (pRBC definition): Matched survival curves.

Kaplan-Meier curves displaying survival probability in the matched sensitivity cohort, comparing UMT (≥15 units of intraoperative pRBC) with MT (10-15 units of intraoperative pRBC). Log-rank *p*-values are provided in each panel. (A) 90-day patient survival: log-rank $p = 0.307$. (B) 3-year patient survival: log-rank $p = 0.414$. (C) Overall patient survival: log-rank $p = 0.585$. (D) 90-day graft survival: log-rank $p = 0.420$. (E) 3-year graft survival: log-rank $p = 0.974$. (F) Overall graft survival: log-rank $p = 0.871$.

(A)

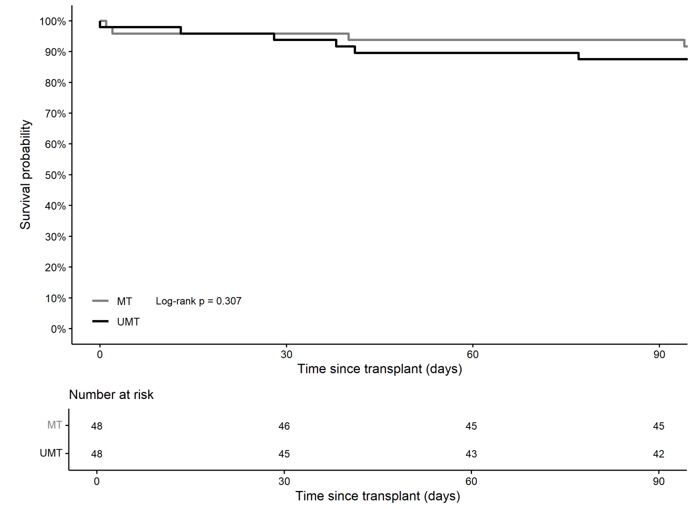

(D)

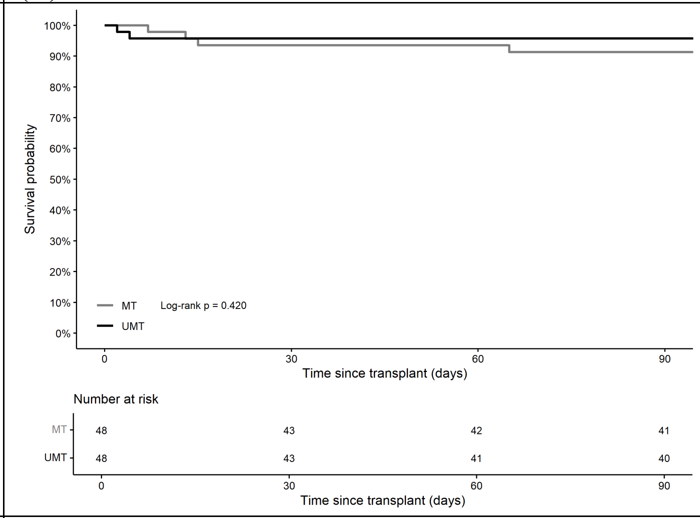

(B)

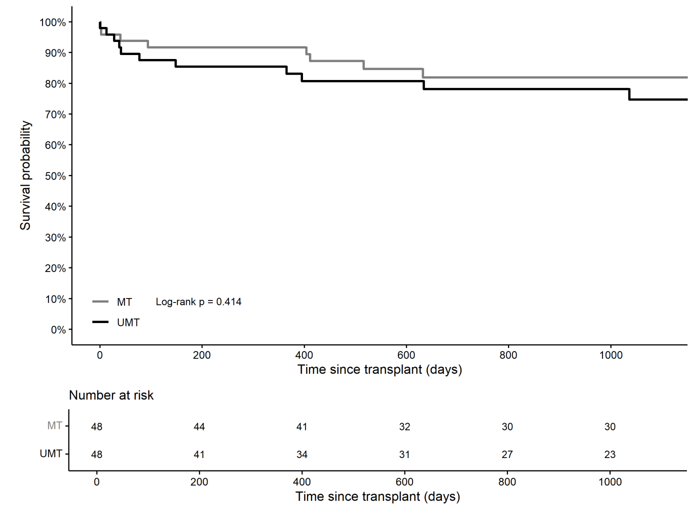

(E)

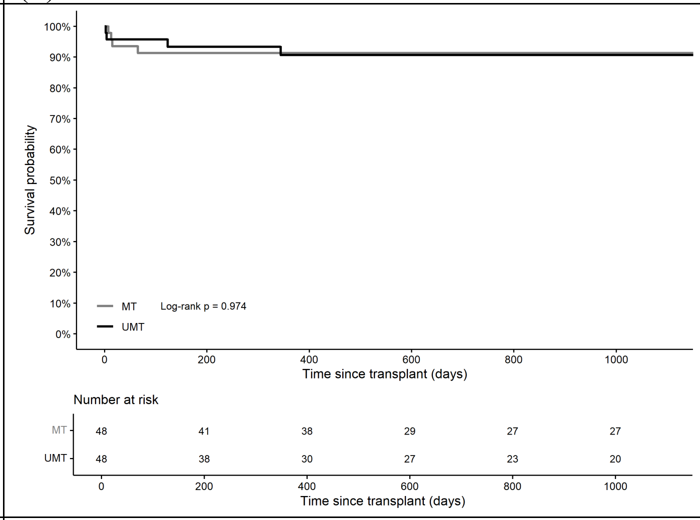

(C)

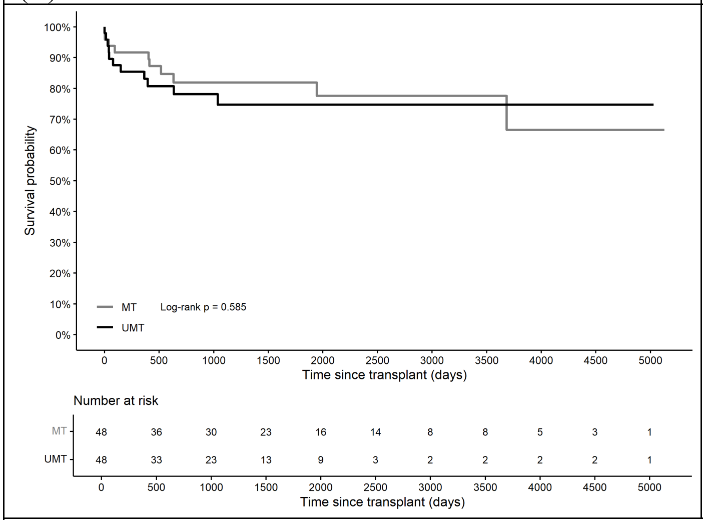

(F)

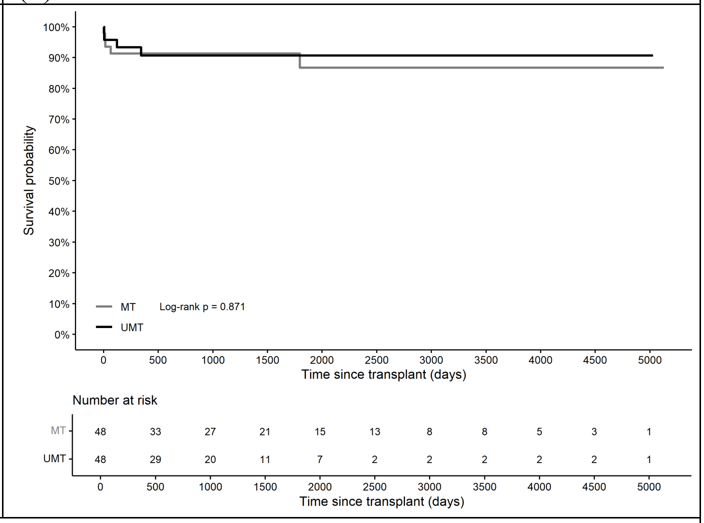

Abbreviations: pRBC; packed red blood cells.
