# Supplementary material for: Effects of massive transfusion (10-20 litres) versus ultramassive transfusion (≥20 litres) on mortality in adult liver transplant recipients: A propensity-score matched study
Source: PLoS One. 2026 May 21;21(5):e0349795. doi: 10.1371/journal.pone.0349795 (PMC13193539; doi:10.1371/journal.pone.0349795)
Supplement: S1 Table — (PDF) [file pone.0349795.s006.pdf]

Supplementary Table 1. Unmatched analysis: Summary of missing data by variable.

**Variable**	**Missing Count**	**Missing Rate (%)**
Baseline fibrinogen clauss	139	45.42
Baseline AST	117	38.24
Donor AST	98	32.03
Baseline APTT	89	29.08
Baseline eGFR	42	13.73
Baseline glucose	28	9.15
Donor cause of death	23	7.52
Donor bilirubin	19	6.21
Donor ALP	17	5.56
Baseline Cl	16	5.23
Donor GGT	15	4.90
Donor ALT	14	4.58
Baseline Mg	11	3.59
MELD-Na	10	3.27
Baseline phosphate	10	3.27
Baseline PT	9	2.94
Baseline ascites calc	7	2.29
Baseline Ca	5	1.63
Baseline ALT	4	1.31
Baseline WCC	4	1.31
Cold ischaemia time	4	1.31
Warm ischaemia time	4	1.31
Baseline platelets	3	0.98
Baseline INR	3	0.98
Baseline K	3	0.98
Baseline albumin	2	0.65
Baseline total protein	2	0.65
Baseline ALP	2	0.65
Baseline GGT	2	0.65
Baseline bilirubin	2	0.65
Baseline Na	1	0.33
Baseline urea	1	0.33
Baseline creatinine	1	0.33

Total ischaemia time	1	0.33
FMS: Platelets	1	0.33
FMS: Autologous wash cells	1	0.33
AKI	1	0.33
AKI stage	1	0.33
Values are reported as frequencies and percentages. **Abbreviations**: ALP, alkaline phosphatase; ALT, alanine aminotransferase; APTT, activated partial thromboplastin time; AST, aspartate transaminase; eGFR, estimated glomerular filtration rate; GGT, gamma-glutamyl transferase; INR, international normalised ratio; MELD-Na, Model for End-Stage Liver Disease sodium-corrected variant; PT, prothrombin time; WCC, white cell count.		
