# Supplementary material for: Effects of massive transfusion (10-20 litres) versus ultramassive transfusion (≥20 litres) on mortality in adult liver transplant recipients: A propensity-score matched study
Source: PLoS One. 2026 May 21;21(5):e0349795. doi: 10.1371/journal.pone.0349795 (PMC13193539; doi:10.1371/journal.pone.0349795)
Supplement: S2 Table — (PDF) [file pone.0349795.s007.pdf]

Supplementary Table 2. Primary analysis: Evaluation of covariate balance across multiple propensity score matching strategies.

**Covariate**	**Unmatched**	**Optimal 1:1**	**Optimal 2:1**	**NNM 1:1**	**NNM 2:1**	**NNM 1:1 caliper 0.1**	**NNM 1:1 caliper 0.2**	**NNM 2:1 caliper 0.1**	**NNM 2:1 caliper 0.2**
Age	0.071	0.019	0.021	0.016	0.021	0.045	0.050	0.129*	0.115*
Sex: male	0.160	0.067	0.146*	0.067	0.146*	0.026	0.025	0.026	0.050
BMI	0.146	0.038	0.092	0.088	0.092	0.014	0.050	0.039	0.018
Transplant indication: chronic liver disease	0.084	0.000	0.021	0.021	0.021	0.074	0.071	0.012	0.035
Transplant indication: cancer	0.156	0.073	0.146*	0.098	0.146*	0.057	0.081	0.014	0.013
Transplant indication: acute liver failure	0.328	0.074	0.111*	0.074	0.111*	0.086	0.082	0.086	0.082
Transplant indication: metabolic disease	0.098	0.061	0.091	0.061	0.091	0.000	0.000	0.000	0.033
Transplant indication: other	0.135	0.099	0.132*	0.099	0.132*	0.000	0.073	0.019	0.055
Transplant indication: re-transplantation	0.065	0.000	0.053	0.000	0.053	0.000	0.058	0.031	0.058
MELD-3	0.060	0.021	0.038	0.050	0.038	0.100	0.088	0.013	0.032
Baseline albumin	0.144	0.100	0.124*	0.114*	0.124*	0.189*	0.147*	0.140*	0.097
Baseline platelets	0.244	0.039	0.224*	0.020	0.224*	0.047	0.052	0.042	0.026

Donor Risk Index	0.170	0.020	0.164*	0.024	0.164*	0.041	0.040	0.049	0.051
Cold ischaemia time	0.251	0.025	0.220*	0.033	0.220*	0.035	0.004	0.032	0.012
Partial graft	0.125	0.061	0.091	0.061	0.091	0.070	0.067	0.070	0.067
Donor pathway: DCD	0.126	0.000	0.108*	0.036	0.108*	0.042	0.080	0.042	0.000

Standardised mean differences (SMDs) for all baseline covariates before and after matching, comparing ultramassive transfusion (≥20 L of intraoperative fluids) with massive transfusion (10–20 L). Multiple matching configurations were evaluated, and the configuration achieving the best overall covariate balance was selected for the primary analysis. *SMD > 0.1. **Abbreviations**: BMI, body mass index; DCD, donation after cardiac death; MELD-3, Model for End-Stage Liver Disease 3.0; NNM, nearest-neighbour matching.
