# Supplementary material for: Effects of massive transfusion (10-20 litres) versus ultramassive transfusion (≥20 litres) on mortality in adult liver transplant recipients: A propensity-score matched study
Source: PLoS One. 2026 May 21;21(5):e0349795. doi: 10.1371/journal.pone.0349795 (PMC13193539; doi:10.1371/journal.pone.0349795)
Supplement: S3 Table — (PDF) [file pone.0349795.s008.pdf]

Supplementary Table 3. Primary analysis: Complete baseline recipient, donor and intraoperative characteristics before and after matching.

	Unmatched (*n* = 306)				Matched (*n* = 188)			
**Variable**	**UMT**	**MT**	***p***	**SMD**	**UMT**	**MT**	***p***	**SMD**
Age (years)	58.2 [49.7–63.8]	56.6 [49.5–63.3]	0.604	0.071	57.8 [49.5–63.6]	56.9 [49.7–62.3]	0.757	0.019
Sex: male, n (%)	63 (66.3)	154 (73)	0.276	0.160†	62 (66)	65 (69.1)	0.760	0.067
Height (cm)	170.5 [165.0–178.0]	172.0 [166.0–178.0]	0.380	–	170.8 [165.0–178.0]	171.0 [165.8–177.8]	0.846	–
Weight (Kg)	84.0 [71.5–96.5]	81.5 [69.2–99.5]	0.658	–	84.3 [71.9–96.8]	81.8 [68.6–101.0]	0.989	–
BMI (Kg/m^2)	28.8 [24.8–32.7]	27.6 [24.0–32.3]	0.364	0.146†	28.8 [24.8–32.8]	28.1 [24.5–33.6]	0.871	0.038
**Transplantation indication**								
Chronic liver disease, n (%)	51 (53.7)	102 (48.3)	0.326	0.084	50 (53.2)	50 (53.2)	0.913	0.000
Cancer	24 (25.3)	67 (31.8)	–	0.156†	24 (25.5)	27 (28.7)	–	0.073
Acute liver failure	2 (2.1)	14 (6.6)	–	0.328†	2 (2.1)	3 (3.2)	–	0.074
Metabolic disease	3 (3.2)	3 (1.4)	–	0.098	3 (3.2)	2 (2.1)	–	0.061
Other	11 (11.6)	17 (8.1)	–	0.135†	11 (11.7)	8 (8.5)	–	0.099
Chronic liver disease, n (%)	4 (4.2)	8 (3.8)	–	0.065	4 (4.3)	4 (4.3)	–	0.000
**Preoperative clinical and laboratory characteristics**								
MELD	19.0 [13.0–28.0]	21.0 [15.4–28.0]	0.226	–	19.0 [13.2–28.0]	19.3 [16.0–26.0]	>0.999	–
MELD-Na	23.5 +/- 8.7	24.0 +/- 8.3	0.628	–	23.6 ± 8.7	23.3 ± 7.9	0.745	–
MELD-3	21.8 [15.1–28.9]	22.3 [16.6–29.0]	0.453	0.060	21.9 [15.4–29.0]	22.3 [15.7–28.1]	0.754	0.021
GEMA	21.7 [16.0–30.5]	22.8 [17.3–29.9]	0.565	–	21.7 [16.2–30.6]	22.4 [15.9–29.4]	0.749	–
Baseline albumin (g/L)	29.0 [26.0–33.0]	31.0 [26.0–36.0]	0.234	0.144†	29.0 [26.0–33.0]	31.0 [26.2–35.8]	0.448	0.100†
Baseline fibrinogen clauss (g/L)	1.8 [1.2–2.8]	1.5 [1.0–2.2]	0.115	–	1.8 [1.2–2.8]	1.5 [1.1–2.3]	0.955	–
Baseline total protein (g/L)	65.0 [55.0–70.0]	62.0 [54.0–68.0]	0.122	–	64.5 [55.0–70.0]	62.0 [55.2–68.0]	0.256	–
Baseline ALT (IU/L)	32.0 [22.0–60.0]	41.0 [26.0–67.5]	0.039*	–	32.0 [22.0–60.0]	37.0 [25.0–57.5]	0.537	–
Baseline AST (IU/L)	58.0 [36.0–96.0]	71.0 [43.0–129.2]	0.117	–	57.0 [36.0–96.8]	65.0 [42.0–101.5]	0.700	–
Baseline GGT (IU/L)	80.0 [43.5–161.5]	56.0 [31.0–122.0]	0.055	–	79.5 [42.8–155.8]	51.5 [32.0–120.2]	0.053	–
Baseline Hb (×10^9/L)	90.0 [78.0–107.5]	86.0 [78.0–104.5]	0.715	–	89.0 [78.0–107.8]	85.0 [78.0–101.8]	0.308	–

Baseline WCC (×10^9/L)	5.4 [3.6–7.9]	5.0 [3.5–7.0]	0.279	–	5.3 [3.6–7.9]	5.0 [3.5–7.0]	0.455	–
Baseline platelets (×10^9/L)	72.0 [49.0–115.5]	65.0 [48.0–95.0]	0.122	0.244†	71.5 [49.0–113.2]	73.0 [52.2–118.5]	0.871	0.039
Baseline PT (sec)	17.3 [14.9–23.0]	18.3 [15.4–24.0]	0.433	–	17.5 [15.0–23.0]	18.0 [15.2–22.5]	0.528	–
Baseline INR	1.6 [1.3–2.1]	1.7 [1.4–2.2]	0.280	–	1.6 [1.3–2.1]	1.6 [1.4–2.0]	0.478	–
Baseline APTT (sec)	39.0 [30.0–53.0]	42.0 [33.8–52.0]	0.217	–	39.0 [30.0–53.0]	43.5 [34.0–51.0]	0.834	–
Baseline ascites calculation	69 (75)	159 (76.8)	0.769	–	68 (74.7)	68 (74.7)	>0.999	–
Baseline bilirubin (μmol/ L)	57.0 [23.0–337.5]	81.0 [34.0–264.0]	0.240	–	58.0 [23.0–341.8]	74.5 [30.2–276.8]	0.845	–
Baseline Ca (mmol/L)	2.2 [2.1–2.4]	2.2 [2.1–2.3]	0.539	–	2.2 [2.1–2.4]	2.2 [2.1–2.3]	0.562	–
Baseline Cl (mmol/L)	102.0 [97.2–105.0]	102.0 [97.0–106.0]	0.743	–	101.6 ± 6.3	100.6 ± 6.7	0.341	–
Baseline K (mmol/L)	4.2 [3.8–4.6]	4.1 [3.8–4.6]	0.188	–	4.2 [3.8–4.6]	4.2 [3.8–4.6]	0.286	–
Baseline Mg (mmol/L)	0.8 [0.7–0.9]	0.8 [0.7–0.9]	0.274	–	0.8 [0.7–0.9]	0.8 [0.7–0.9]	0.535	–
Baseline Na (mmol/L)	136.0 [133.0–137.5]	136.0 [132.0–139.8]	0.298	–	136.0 [133.0–137.0]	136.5 [132.0–139.0]	0.728	–
Baseline phosphate (mmol/L)	1.1 [1.0–1.3]	1.1 [0.9–1.3]	0.286	–	1.1 [1.0–1.3]	1.1 [1.0–1.3]	0.289	–
Baseline glucose (mmol/L)	6.2 [5.1–9.0]	6.4 [5.4–8.1]	0.936	–	6.1 [5.1–9.0]	6.0 [5.2–7.6]	0.416	–
Baseline urea (mmol/L)	8.2 [5.3–13.4]	8.9 [5.4–13.8]	0.902	–	8.1 [5.3–13.1]	8.6 [5.5–12.6]	0.785	–
Baseline creatinine (μmol/ L)	96.0 [70.5–137.0]	104.5 [72.2–141.0]	0.552	–	96.0 [70.2–137.0]	92.5 [71.0–127.0]	0.484	–
Baseline eGFR (mL/min/1.73 m^2)	69.0 [48.8–90.0]	61.5 [44.0–90.0]	0.451	–	69.0 [48.5–90.0]	80.0 [49.0–90.0]	0.721	–
**Donor and graft characteristics**								
Donor age (years)	47.0 [32.0–57.0]	45.0 [32.0–58.0]	0.806	–	47.0 [32.0–57.0]	50.5 [36.0–58.8]	0.480	–
Donor pathway: DCD, n (%)	9 (9.5)	14 (6.6)	0.482	0.126†	9 (9.6)	9 (9.6)	>0.999	0.000
Donor bilirubin (μmol/l)	10.0 [6.0–16.5]	9.0 [6.0–13.0]	0.241	–	10.0 [6.0–15.8]	9.0 [7.0–13.0]	0.417	–
Donor ALP (IU/L)	73.0 [60.0–87.0]	75.0 [59.0–96.0]	0.338	–	73.0 [59.8–87.2]	74.0 [59.0–96.5]	0.111	–
Donor ALT (IU/L)	34.0 [17.2–89.8]	36.5 [20.2–86.8]	0.476	–	34.0 [17.0–90.0]	38.0 [21.5–84.5]	0.721	–
Donor AST (IU/L)	42.0 [21.0–70.5]	43.5 [22.0–80.5]	0.730	–	42.0 [21.0–70.5]	41.0 [18.5–77.5]	0.691	–
Donor GGT (IU/L)	38.0 [18.2–67.8]	45.0 [22.0–83.0]	0.136	–	38.0 [18.2–67.8]	45.0 [20.0–83.5]	0.038*	–
**Donor cause of death, n (%)**								
Stroke	49 (57.6)	100 (50.5)	0.580	–	49 (58.3)	51 (60.7)	0.734	–
Trauma	9 (10.6)	27 (13.6)			8 (9.5)	7 (8.3)		

Anoxia	20 (23.5)	58 (29.3)			20 (23.8)	20 (23.8)		
Other	7 (8.2)	13 (6.6)			7 (8.3)	6 (7.1)		
Donor national share	18 (18.9)	23 (10.9)	0.069	–	18 (19.1)	11 (11.7)	0.265	–
Donor Risk Index (DRI)	1.4 [1.3–1.8]	1.4 [1.1–1.7]	0.189	0.170†	1.4 [1.3–1.8]	1.5 [1.2–1.8]	0.801	0.020
**Organ transplantation, n (%)**				0.125†				0.061
Partial graft	4 (4.2)	11 (5.2)	>0.999		3 (3.2)	4 (4.3)	>0.999	
Intestinal transplant	1 (1.1)	2 (0.9)	>0.999		1 (1.1)	1 (1.1)	>0.999	
Kidney transplant	4 (4.2)	11 (5.2)	>0.999		4 (4.3)	4 (4.3)	>0.999	
Other organ transplant	1 (1.1)	2 (0.9)	>0.999		1 (1.1)	1 (1.1)	>0.999	
Multiorgan transplant	5 (5.3)	13 (6.2)	>0.999		5 (5.3)	5 (5.3)	>0.999	
**Intraoperative characteristics**								
Cold ischaemia time (min)	376.5 [287.5–447.8]	331.0 [271.8–407.0]	0.032*	0.251†	370.9 ± 110.2	368.1 ± 96.1	0.855	0.025
Warm ischaemia time (min)	48.0 [40.2–56.8]	45.0 [40.0–51.2]	0.051	–	48.0 [40.2–56.8]	47.0 [41.0–53.0]	0.086	–
Total ischaemia time (min)	431.0 [332.5–501.5]	376.5 [316.0–460.0]	0.008*	–	425.7 ± 114.7	416.0 ± 97.4	0.529	–
**Intraoperative transfusion and fluid**								
Albumex (L)	2.5 [2.1–3.6]	1.3 [1.1–1.6]	<0.001*	–	2.5 [2.1–3.6]	1.3 [1.1–1.6]	<0.001*	–
Plasmalyte (L)	14.0 [11.0–18.5]	7.0 [6.0–8.0]	<0.001*	–	14.0 [11.0–18.0]	7.0 [6.0–8.0]	<0.001*	–
Packed red blood cells (unit)	14.0 [10.7–20.0]	6.3 [4.0–10.0]	<0.001*	–	14.0 [10.6–19.5]	6.8 [4.2–10.0]	<0.001*	–
Fresh frozen plasma (unit)	6.0 [4.0–8.0]	2.0 [1.0–4.0]	<0.001*	–	6.0 [4.0–8.0]	2.0 [1.0–4.0]	<0.001*	–
Platelets (unit)	3.0 [2.0–4.0]	1.0 [1.0–2.0]	<0.001*	–	3.0 [2.0–4.0]	1.5 [1.0–2.0]	<0.001*	–
Cryoprecipitate (unit)	15.0 [9.0–21.0]	7.0 [0.0–10.0]	<0.001*	–	15.0 [8.5–21.0]	7.0 [0.0–10.0]	<0.001*	–
Autologous washed cells (L)	7.1 [5.2–11.3]	2.6 [1.8–3.6]	<0.001*	–	7.1 [5.2–11.3]	2.5 [1.8–3.4]	<0.001*	–
Washed donor blood (L)	0.0 [0.0–900.0]	0.0 [0.0–900.0]	0.664	–	0.0 [0.0–900.0]	0.0 [0.0–850.0]	0.407	–
Allogenic red blood cells (L)	3.4 [2.6–4.8]	1.6 [1.0–2.1]	<0.001*	–	3.4 [2.5–4.8]	1.6 [1.0–2.1]	<0.001*	–
Total volume (L)	27.4 [22.6–39.1]	13.2 [11.2–15.5]	<0.001*	–	27.2 [22.6–38.3]	13.1 [11.1–15.5]	<0.001*	–

Continuous variables are presented as mean ± standard deviation or median [interquartile range]. Categorical variables are presented as frequencies (percentages). For unmatched comparisons, the independent t-test, Mann-Whitney U test, chi-square test, and Fisher's exact test were used. For matched comparisons, the paired t-test, Wilcoxon signed-rank test, McNemar's test, exact McNemar test, and Stuart-Maxwell test were used. Non-parametric tests were used if regression did not converge; descriptive statistics were reported if comparison was infeasible. *$p < 0.05$ indicates statistical significance. †SMD > 0.1. **Abbreviations**: ALP, alkaline phosphatase; ALT, alanine aminotransferase; APTT, activated partial thromboplastin time; AST, aspartate transaminase; BMI, body mass index; DCD, donation after cardiac

death; eGFR, estimated glomerular filtrate rate; FMS, fluid management system; GEMA, Gender-Equity Model for Liver Allocation; GGT, gamma-glutamyl transferase; INR, international normalised ratio; MELD, Model for End-Stage Liver Disease; MELD-3, Model for End-Stage Liver Disease 3.0; MELD-Na, Model for End-Stage Liver Disease sodium-corrected variant; MT, massive transfusion; PT, prothrombin time; SMD, standardised mean difference; UMT, ultramassive transfusion; WCC, white cell count
