# Supplementary material for: Effects of massive transfusion (10-20 litres) versus ultramassive transfusion (≥20 litres) on mortality in adult liver transplant recipients: A propensity-score matched study
Source: PLoS One. 2026 May 21;21(5):e0349795. doi: 10.1371/journal.pone.0349795 (PMC13193539; doi:10.1371/journal.pone.0349795)
Supplement: S4 Table — (PDF) [file pone.0349795.s009.pdf]

Supplementary Table 4. Unmatched analysis: Perioperative and long-term outcomes.

Outcome	Unmatched (*n* = 306)			
	**UMT**	**MT**	**Effect size (95% CI)**	***p***
**Mortality**				
90-day mortality, n (%)	11 (11.6)	2 (0.9)	13.68 (3.58–89.66)	<0.001*
3-year mortality, n (%)	20 (21.1)	16 (7.6)	3.25 (1.60–6.69)	0.001*
Overall mortality, n (%)	27 (28.4)	33 (15.6)	2.14 (1.19–3.83)	0.010*
**Graft outcomes**				
PNF, n (%)	5 (5.3)	4 (1.9)	2.87 (0.74–11.85)	0.122
EAD, n (%)	41 (43.2)	61 (28.9)	1.87 (1.13–3.09)	0.015*
90-day graft loss, n (%)	6 (6.3)	10 (4.7)	1.36 (0.45–3.76)	0.568
3-year graft loss, n (%)	10 (10.5)	11 (5.2)	2.14 (0.86–5.26)	0.095
Overall graft loss, n (%)	10 (10.5)	14 (6.6)	1.66 (0.69–3.85)	0.245
**Thrombotic complications**				
**Hepatic artery thrombosis (HAT)**				
30-day HAT, n (%)	1 (1.1)	6 (2.8)	0.36 (0.02–2.17)	0.352
Overall HAT, n (%)	3 (3.2)	7 (3.3)	0.95 (0.20–3.50)	0.942
**Portal vein thrombosis (PVT)**				
30-day PVT, n (%)	1 (1.1)	3 (1.4)	0.74 (0.04–5.85)	0.793
Overall PVT, n (%)	5 (5.3)	5 (2.4)	2.29 (0.62–8.42)	0.199
**Hepatic vein thrombosis (HVT)**				
30-day HVT, n (%)	0 (0.0)	1 (0.5)	– §	>0.999
Overall HVT, n (%)	0 (0.0)	1 (0.5)	– §	>0.999
**Composite thrombosis**				
30-day HAT or PVT, n (%)	2 (2.1)	9 (4.3)	0.48 (0.07–1.92)	0.358
Overall HAT or PVT, n (%)	8 (8.4)	12 (5.7)	1.52 (0.58–3.82)	0.374
30-day other thrombosis, n (%)	3 (3.2)	3 (1.4)	2.26 (0.41–12.42)	0.323
Overall other thrombosis, n (%)	6 (6.3)	9 (4.3)	1.51 (0.49–4.32)	0.445
30-day any thrombosis, n (%)	5 (5.3)	12 (5.7)	0.92 (0.29–2.56)	0.881
Overall any thrombosis, n (%)	13 (13.7)	21 (10.0)	1.43 (0.67–2.97)	0.338
**Renal outcomes**				
Acute kidney injury (AKI), n (%)	65 (68.4)	156 (74.3)	0.75 (0.44–1.28)	0.289
**AKI Stage distribution**, n (%)				
No AKI	30 (31.6)	55 (26.2)	-0.06 (-0.20–0.08)	0.384
Stage 1	42 (44.2)	99 (47.1)		
Stage 2	15 (15.8)	36 (17.1)		
Stage 3	8 (8.4)	20 (9.5)		
LOS (days), median [IQR]	29.0 [14.0–53.0]	23.0 [14.0–39.0]	0.12 (-0.02–0.25)	0.097

Continuous variables are presented as mean ± standard deviation or median [interquartile range]. Categorical variables are presented as frequencies (percentages). The independent t-test or Mann-Whitney U test was used for continuous or ordinal outcomes. The chi-squared test, Fisher's exact test, or logistic regression was used for binary outcomes. Non-parametric tests were used if regression did not converge; descriptive statistics were reported if comparison was infeasible. *$p < 0.05$ indicates statistical significance. §Effect size or p-value not estimable due to sparse data or zero-cell counts. Effect sizes are reported as odds ratios for binary outcomes and rank biserial r for ordinal or continuous outcomes. **Abbreviations**: CI, confidence interval; EAD, early allograft dysfunction; LOS, length of stay; MT, massive transfusion; PNF, primary non-function; UMT, ultramassive transfusion.
