# Supplementary material for: Effects of massive transfusion (10-20 litres) versus ultramassive transfusion (≥20 litres) on mortality in adult liver transplant recipients: A propensity-score matched study
Source: PLoS One. 2026 May 21;21(5):e0349795. doi: 10.1371/journal.pone.0349795 (PMC13193539; doi:10.1371/journal.pone.0349795)
Supplement: S5 Table — (PDF) [file pone.0349795.s010.pdf]

Supplementary Table 5. Unmatched analysis: Cox proportional hazards regression for patient and graft survival.

**Outcome**	**HR (95% CI)**	***p***	**PH Global *p***
**Patient survival**			
90-day survival	12.84 (2.85–57.94)	<0.001*	0.703
3-year survival	3.13 (1.62–6.04)	<0.001*	0.356
Overall survival	2.34 (1.41–3.91)	0.001*	0.455
**Graft survival**			
90-day survival	1.39 (0.51–3.84)	0.520	0.220
3-year survival	2.15 (0.91–5.06)	0.080	0.640
Overall survival	1.81 (0.80–4.08)	0.155	0.752

Univariate Cox proportional hazards regression models were used to estimate hazard ratios (HR), 95% confidence intervals (CI), and *p*-values comparing ultramassive transfusion (≥20 L of intraoperative fluids) with massive transfusion (10–20 L) across 90-day, 3-year, and overall follow-up periods. The proportional hazards assumption was verified using Schoenfeld residuals and held for all reported models (PH Global $p > 0.05$). *$p < 0.05$ indicates statistical significance. **Abbreviations**: CI, confidence interval; HR, hazard ratio; PH, proportional hazards.
