# Supplementary material for: Effects of massive transfusion (10-20 litres) versus ultramassive transfusion (≥20 litres) on mortality in adult liver transplant recipients: A propensity-score matched study
Source: PLoS One. 2026 May 21;21(5):e0349795. doi: 10.1371/journal.pone.0349795 (PMC13193539; doi:10.1371/journal.pone.0349795)
Supplement: S6 Table — (PDF) [file pone.0349795.s011.pdf]

Supplementary Table 6. Sensitivity analysis I (expanded comparator): Evaluation of covariate balance.

**Covariate**	**Unmatched (*n* = 868)**	**Matched (*n* = 376)**
Age (years)	0.252	0.091
Sex: male, n (%)	0.037	0.037
BMI	0.273	0.012
Transplant indication: chronic liver disease/cirrhosis	0.170	0.043
Transplant indication: cancer	0.206	0.057
Transplant indication: acute liver failure	0.381	0.074
Transplant indication: metabolic disease	0.042	0.081
Transplant indication: other	0.171	0.022
Transplant indication: re-transplantation	0.013	0.070
MELD-3	0.033	0.077
Baseline albumin	0.062	0.065
Baseline platelets	0.026	0.003
Donor Risk Index (DRI)	0.063	0.004
Cold Ischaemia Time	0.334	0.019
Partial Graft: Yes, n (%)	0.164	0.101
Donation Pathway: DCD, n (%)	0.066	0.000

Standardised mean differences (SMDs) for all baseline covariates before and after propensity score matching, comparing ultramassive transfusion (≥20 L of intraoperative fluids) with non-UMT (<20 L). Covariate balance was assessed using SMDs, with an SMD <0.1 indicating adequate balance. *SMD > 0.1. **Abbreviations**: BMI, body mass index; DCD, donation after cardiac death; MELD-3, Model for End-Stage Liver Disease 3.0.
