# Supplementary material for: Effects of massive transfusion (10-20 litres) versus ultramassive transfusion (≥20 litres) on mortality in adult liver transplant recipients: A propensity-score matched study
Source: PLoS One. 2026 May 21;21(5):e0349795. doi: 10.1371/journal.pone.0349795 (PMC13193539; doi:10.1371/journal.pone.0349795)
Supplement: S7 Table — (PDF) [file pone.0349795.s012.pdf]

Supplementary Table 7. Sensitivity analysis I (expanded comparator): Perioperative and long-term outcomes in the matched sensitivity cohort.

**Outcome**	**Matched (*n* = 376)**			
	**UMT**	**Non-UMT**	**Effect size (95% CI)**	***p***
**Mortality**				
90-day mortality, n (%)	11 (11.7)	3 (1.1)	11.00 (3.07–39.43)	<0.001*
3-year mortality, n (%)	19 (20.2)	17 (6.0)	3.89 (1.91–7.96)	<0.001*
Overall mortality, n (%)	26 (27.7)	36 (12.8)	2.45 (1.41–4.28)	0.002*
**Graft outcomes**				
PNF, n (%)	5 (5.3)	3 (1.1)	5.00 (1.19–20.92)	0.028*
EAD, n (%)	41 (43.6)	86 (30.5)	1.78 (1.09–2.91)	0.021*
90-day graft loss, n (%)	6 (6.4)	7 (2.5)	2.76 (0.88–8.64)	0.082
3-year graft loss, n (%)	10 (10.6)	12 (4.3)	3.00 (1.16–7.76)	0.024*
Overall graft loss, n (%)	10 (10.6)	15 (5.3)	2.23 (0.93–5.34)	0.073
**Thrombotic complications**				
**Hepatic artery thrombosis (HAT)**				
30-day HAT, n (%)	1 (1.1)	2 (0.7)	1.50 (0.14–16.54)	0.741
Overall HAT, n (%)	3 (3.2)	5 (1.8)	1.80 (0.43–7.53)	0.421
**Portal vein thrombosis (PVT)**				
30-day PVT, n (%)	1 (1.1)	4 (1.4)	0.75 (0.08–6.71)	0.797
Overall PVT, n (%)	5 (5.3)	12 (4.3)	1.25 (0.44–3.55)	0.675
**Hepatic vein thrombosis (HVT)**				
30-day HVT, n (%)	0 (0.0)	0 (0.0)	– §	– §
Overall HVT, n (%)	0 (0.0)	0 (0.0)	– §	– §
**Composite thrombosis**				
30-day HAT or PVT, n (%)	2 (2.1)	6 (2.1)	1.00 (0.20–4.95)	>0.999
Overall HAT or PVT, n (%)	8 (8.5)	16 (5.7)	1.50 (0.64–3.50)	0.349
30-day other thrombosis, n (%)	3 (3.2)	3 (1.1)	3.00 (0.61–14.86)	0.178
Overall other thrombosis, n (%)	6 (6.4)	8 (2.8)	2.25 (0.78–6.48)	0.133
30-day any thrombosis, n (%)	5 (5.3)	9 (3.2)	1.78 (0.55–5.74)	0.336
Overall any thrombosis, n (%)	13 (13.8)	24 (8.5)	1.74 (0.84–3.62)	0.136
**Renal outcomes**				
Acute kidney injury (AKI), n (%)	64 (68.1)	203 (72.0)	0.82 (0.48–1.39)	0.456
**AKI Stage distribution**, n (%)				
No AKI	30 (31.9)	25 (26.6)	-0.13 (-0.39–0.15)	0.337
Stage 1	41 (43.6)	38 (40.4)		
Stage 2	15 (16)	23 (24.5)		

Stage 3	8 (8.5)	8 (8.5)		
LOS (days), median [IQR]	29.0 [14.0–53.0]	16.0 [12.0–24.8]	0.48 (0.28–0.64)	<0.001*

Continuous variables are presented as mean ± standard deviation or median [interquartile range]. Categorical variables are presented as frequencies (percentages). The paired t-test or the Wilcoxon signed-rank test was used for continuous or ordinal outcomes. The McNemar's test, McNemar's exact test, or conditional logistic regression was used for binary outcomes. Non-parametric tests were used if regression did not converge; descriptive statistics were reported if comparison was infeasible. $^*p < 0.05$ indicates statistical significance. §Effect size or *p*-value not estimable due to sparse data or zero-cell counts. Effect sizes are reported as odds ratios for binary outcomes and rank biserial *r* for ordinal or continuous outcomes. **Abbreviations**: CI, confidence interval; EAD, early allograft dysfunction; LOS, length of stay; PNF, primary non-function; UMT, ultramassive transfusion.
