# Supplementary material for: Effects of massive transfusion (10-20 litres) versus ultramassive transfusion (≥20 litres) on mortality in adult liver transplant recipients: A propensity-score matched study
Source: PLoS One. 2026 May 21;21(5):e0349795. doi: 10.1371/journal.pone.0349795 (PMC13193539; doi:10.1371/journal.pone.0349795)
Supplement: S8 Table — (PDF) [file pone.0349795.s013.pdf]

**Supplementary Table 8.** Sensitivity analysis I (expanded comparator): Conditional Cox proportional hazards regression for patient and graft survival in the matched sensitivity cohort.

| **Outcome** | **HR (95% CI)** | ***p*** | **PH Global *p*** |
|---|---|---|---|
| **Patient survival** | | | |
| 90-day survival | 11.00 (3.07–39.43) | <0.001* | 0.360 |
| 3-year survival | 4.28 (2.11–8.67) | <0.001* | 0.251 |
| Overall survival | 2.92 (1.61–5.28) | <0.001* | 0.029* |
| **Graft survival** | | | |
| 90-day survival | 2.49 (0.84–7.43) | 0.101 | 0.309 |
| 3-year survival | 3.06 (1.24–7.56) | 0.015* | 0.623 |
| Overall survival | 2.77 (1.15–6.67) | 0.023* | 0.385 |

Univariate Cox proportional hazards regression models were used to estimate hazard ratios (HR), 95% confidence intervals (CI), and *p*-values comparing ultramassive transfusion (≥20 L of intraoperative fluids) with non-UMT (<20 L) across 90-day, 3-year, and overall follow-up periods. The proportional hazards assumption was verified using Schoenfeld residuals. The proportional hazards assumption was violated for the overall patient survival model (PH Global $p = 0.029$); the reported HR is presented as the average effect over the follow-up period. *$p < 0.05$ indicates statistical significance. **Abbreviations**: CI, confidence interval; HR, hazard ratio; PH, proportional hazards.
