# Supplementary material for: Effects of massive transfusion (10-20 litres) versus ultramassive transfusion (≥20 litres) on mortality in adult liver transplant recipients: A propensity-score matched study
Source: PLoS One. 2026 May 21;21(5):e0349795. doi: 10.1371/journal.pone.0349795 (PMC13193539; doi:10.1371/journal.pone.0349795)
Supplement: S9 Table — (PDF) [file pone.0349795.s014.pdf]

Supplementary Table 9. Sensitivity analysis II (pRBC exposure): Covariate balance assessment.

**Covariate**	**Unmatched (*n* = 142)**	**Matched (*n* = 96)**
Age (years)	0.008	0.033
Sex: male, n (%)	0.201*	0.087
BMI	0.003	0.070
Transplant indication: chronic liver disease/cirrhosis	0.121*	0.042
Transplant indication: cancer	0.101*	0.057
Transplant indication: acute liver failure	0.129*	0.081
Transplant indication: metabolic disease	0.315*	0.000
Transplant indication: other	0.167*	0.063
Transplant indication: re-transplantation	0.052	0.000
MELD-3	0.002	0.037
Baseline albumin	0.003	0.032
Baseline platelets	0.218*	0.038
Donor Risk Index (DRI)	0.148*	0.090
Cold Ischaemia Time	0.283*	0.129*
Partial Graft: Yes, n (%)	0.216*	0.157*
Donation Pathway: DCD, n (%)	0.046	0.000

Standardised mean differences (SMDs) for all baseline covariates before and after propensity score matching in the pRBC-centred sensitivity analysis, comparing UMT (≥15 units of intraoperative pRBC) with MT (10–14 units). Covariate balance was assessed using SMDs, with an SMD <0.1 indicating adequate balance. *SMD > 0.1.
Abbreviations: BMI, body mass index; DCD, donation after cardiac death; MELD-3, Model for End-Stage Liver Disease 3.0; pRBC, packed red blood cells.
