# Supplementary material for: Effects of massive transfusion (10-20 litres) versus ultramassive transfusion (≥20 litres) on mortality in adult liver transplant recipients: A propensity-score matched study
Source: PLoS One. 2026 May 21;21(5):e0349795. doi: 10.1371/journal.pone.0349795 (PMC13193539; doi:10.1371/journal.pone.0349795)
Supplement: S10 Table — (PDF) [file pone.0349795.s015.pdf]

Supplementary Table 10. Sensitivity analysis II (pRBC exposure): Perioperative and long-term outcomes in the matched sensitivity cohort.

Outcome	Matched (*n* = 96)			
	**UMT**	**MT**	**Effect size (95% CI)**	***p***
**Mortality**				
90-day mortality, n (%)	6 (12.5)	3 (6.2)	2.00 (0.50–8.00)	0.327
3-year mortality, n (%)	11 (22.9)	8 (16.7)	1.43 (0.54–3.75)	0.469
Overall mortality, n (%)	11 (22.9)	10 (20.8)	1.14 (0.41–3.15)	0.796
**Graft outcomes**				
PNF, n (%)	2 (4.2)	2 (4.2)	1.00 (0.14–7.10)	>0.999
EAD, n (%)	20 (41.7)	20 (41.7)	1.00 (0.46–2.16)	>0.999
90-day graft loss, n (%)	2 (4.2)	4 (8.3)	0.50 (0.09–2.73)	0.423
3-year graft loss, n (%)	4 (8.3)	4 (8.3)	1.00 (0.25–4.00)	>0.999
Overall graft loss, n (%)	4 (8.3)	5 (10.4)	0.80 (0.21–2.98)	0.739
**Thrombotic complications**				
**Hepatic artery thrombosis (HAT)**				
30-day HAT, n (%)	1 (2.1)	0 (0.0)	– §	>0.999
Overall HAT, n (%)	2 (4.2)	0 (0.0)	– §	>0.999
**Portal vein thrombosis (PVT)**				
30-day PVT, n (%)	0 (0.0)	3 (6.2)	– §	>0.999
Overall PVT, n (%)	2 (4.2)	3 (6.2)	0.67 (0.11–3.99)	0.657
**Hepatic vein thrombosis (HVT)**				
30-day HVT, n (%)	0 (0.0)	1 (2.1)	– §	>0.999
Overall HVT, n (%)	0 (0.0)	1 (2.1)	– §	>0.999
**Composite thrombosis**				
30-day HAT or PVT, n (%)	1 (2.1)	3 (6.2)	0.33 (0.03–3.20)	0.341
Overall HAT or PVT, n (%)	4 (8.3)	3 (6.2)	1.33 (0.30–5.96)	0.706
30-day other thrombosis, n (%)	3 (6.2)	0 (0.0)	– §	>0.999
Overall other thrombosis, n (%)	4 (8.3)	2 (4.2)	2.00 (0.37–10.92)	0.423
30-day any thrombosis, n (%)	4 (8.3)	3 (6.2)	1.33 (0.30–5.96)	0.706
Overall any thrombosis, n (%)	7 (14.6)	5 (10.4)	1.50 (0.42–5.32)	0.530
**Renal outcomes**				
Acute kidney injury (AKI), n (%)	36 (75.0)	39 (81.2)	0.73 (0.29–1.81)	0.493
**AKI Stage distribution**, n (%)				
No AKI	12 (25.0)	10 (20.8)	-0.01 (-0.37–0.35)	0.959

Stage 1	25 (52.1)	27 (56.2)		
Stage 2	8 (16.7)	8 (16.7)		
Stage 3	3 (6.2)	3 (6.2)		
LOS (days), median [IQR]	33.5 [21.2–60.2]	33.0 [18.8–47.5]	0.22 (-0.10–0.50)	0.191

Continuous variables are presented as mean ± standard deviation or median [interquartile range]. Categorical variables are presented as frequencies (percentages). The paired t-test or the Wilcoxon signed-rank test was used for continuous or ordinal outcomes. The McNemar's test, McNemar's exact test, or conditional logistic regression was used for binary outcomes. Non-parametric tests were used if regression did not converge; descriptive statistics were reported if comparison was infeasible. *$p < 0.05$ indicates statistical significance. §Effect size or p-value not estimable due to sparse data or zero-cell counts. Effect sizes are reported as odds ratios for binary outcomes and rank biserial r for ordinal or continuous outcomes. **Abbreviations**: CI, confidence interval; EAD, early allograft dysfunction; LOS, length of stay; MT, massive transfusion; PNF, primary non-function; UMT, ultramassive transfusion.
