# Supplementary material for: Effects of massive transfusion (10-20 litres) versus ultramassive transfusion (≥20 litres) on mortality in adult liver transplant recipients: A propensity-score matched study
Source: PLoS One. 2026 May 21;21(5):e0349795. doi: 10.1371/journal.pone.0349795 (PMC13193539; doi:10.1371/journal.pone.0349795)
Supplement: S11 Table — (PDF) [file pone.0349795.s016.pdf]

Supplementary Table 11. Sensitivity analysis II (pRBC exposure): Conditional Cox proportional hazards regression for patient and graft survival in the matched sensitivity cohort

**Outcome**	**HR (95% CI)**	***p***	**PH Global *p***
**Patient survival**			
90-day survival	2.00 (0.50–8.00)	0.327	0.411
3-year survival	1.13 (0.43–2.92)	0.808	0.443
Overall survival	1.13 (0.43–2.92)	0.808	0.443
**Graft survival**			
90-day survival	0.67 (0.11–3.99)	0.657	0.053
3-year survival	1.33 (0.30–5.96)	0.706	0.763
Overall survival	1.33 (0.30–5.96)	0.706	0.763

Univariate Cox proportional hazards regression models were used to estimate hazard ratios (HR), 95% confidence intervals (CI), and *p*-values comparing UMT (≥15 units of intraoperative pRBC) with MT (10–14) for patient and graft survival across 90-day, 3-year, and overall follow-up periods. The 3-year and overall patient survival models produced identical estimates, likely reflecting model instability due to few events across matched strata; these results should be interpreted with caution. The proportional hazards assumption was verified using Schoenfeld residuals and held for all models (PH Global $p > 0.05$). *$p < 0.05$ indicates statistical significance. **Abbreviations**: PH, proportional hazards; pRBC, packed red blood cells.
