# Supplementary material for: Effects of massive transfusion (10-20 litres) versus ultramassive transfusion (≥20 litres) on mortality in adult liver transplant recipients: A propensity-score matched study
Source: PLoS One. 2026 May 21;21(5):e0349795. doi: 10.1371/journal.pone.0349795 (PMC13193539; doi:10.1371/journal.pone.0349795)
Supplement: S12 Table — (PDF) [file pone.0349795.s017.pdf]

Supplementary Table 12. Sensitivity analysis III (time period): Conditional Cox proportional hazards regression for patient survival in the matched cohort

	Early era		Contemporary era		Era-adjusted			
**Outcome**	**UMT (*n* = 34)**	**MT (*n* = 53)**	**UMT (*n* = 60)**	**MT (*n* = 41)**	**HR (95% CI)**	***p***	***p* (interaction)**	**PH Global *p***
90-day mortality, n (%)	5 (14.7)	0 (0.0)	6 (10.0)	0 (0.0)	– §	– §	– §	
3-year mortality, n (%)	8 (23.5)	4 (7.5)	11 (18.3)	3 (7.3)	2.55 (1.06–6.11)	0.036*	0.350	0.423
Overall mortality, n (%)	14 (41.2)	11 (20.8)	12 (20.0)	3 (7.3)	2.54 (1.17–5.49)	0.018*	0.266	0.786

Era-adjusted conditional Cox proportional hazards models were fitted to the matched cohort, with transplant era included as a covariate and matched pair as strata, comparing ultramassive transfusion (UMT; ≥20 L of intraoperative fluids) with massive transfusion (MT; 10–20 L). The early era spans from December 2009 to October 2018, and the contemporary era spans from October 2018 to December 2023, defined by the median transplant date across the unmatched cohort. Hazard ratios (HR), 95% confidence intervals (CI), and *p*-values are reported across 3-year and overall follow-up periods. The interaction *p*-value (likelihood ratio test) tests whether the magnitude of the UMT-mortality association differs between eras. The proportional hazards assumption was verified using Schoenfeld residuals and held for all reported models (PH Global $p > 0.05$). Event counts are presented as n (%) within each era and group. †90-day mortality was excluded from formal analysis because complete separation persisted within both eras, precluding convergence of the era-adjusted Cox model. $p < 0.05$ indicates statistical significance. **Abbreviations**: MT, massive transfusion; PH, proportional hazards; UMT, ultramassive transfusion.
