# Supplementary material for: Effects of massive transfusion (10-20 litres) versus ultramassive transfusion (≥20 litres) on mortality in adult liver transplant recipients: A propensity-score matched study
Source: PLoS One. 2026 May 21;21(5):e0349795. doi: 10.1371/journal.pone.0349795 (PMC13193539; doi:10.1371/journal.pone.0349795)
Supplement: Attachment — Submitted filename: Response_To Reviewers_4April26.pdf [file pone.0349795.s020.pdf]

RESPONSE TO REVIEWERS

Professor Pavel Strnad
Academic Editor
PLOS One

PONE-D-26-07916
Title: Effects of massive transfusion (10-20 litres) versus ultramassive transfusion (≥20 litres) on mortality in adult liver transplant recipients: A propensity-score matched study

Dear Pavel Strnad

My coauthors and I would like to thank you and the expert reviewers for taking the time to provide a very constructive and thoughtful review of our manuscript.

As requested, we have included the following items with our resubmission:

1. A point-by-point response letter (to follow) that addresses each issue raised by the Academic Editor and the Reviewers. I have uploaded this letter as a separate file labelled "**Response to Reviewers**."

2. A marked-up copy of the manuscript that highlights changes made to the original version. This has been uploaded as a separate file labelled "**Revised Manuscript with Tracked Changes highlighted in red**."

3. An unmarked version of our revised paper without tracked changes. This has also been uploaded as a separate file labelled "**Manuscript.**"

Editorial / General Points

1. PLOS ONE style and file naming

Authors' response: We thank the Editors for this important reminder and have carefully reviewed the manuscript and all associated files to ensure compliance with PLOS ONE's formatting and file-naming requirements. All manuscript, figure, and supplementary files have now been renamed and formatted in accordance with the journal's guidelines.

2. Anonymisation of supporting data

Authors' response: We are grateful for the Editors' vigilance regarding patient confidentiality and data governance. Following your advice, we have reviewed the original dataset in detail, removed all directly identifying fields, and ensured that no potentially identifying information remains (including hidden spreadsheet columns). The re-uploaded data file now contains only fully anonymised variables, consistent with the consent framework approved by our Human Research Ethics Committee and applicable local regulations.

3. Citation of suggested references

Authors' response: We thank the Editors and reviewers for their thoughtful reference suggestions. We have systematically reviewed the recommended publications and have cited those

that are directly relevant to our study question, methods, or interpretation, while avoiding citation purely for completeness. Where suggested articles were not clearly applicable to orthotopic liver transplantation (OLT) or to ultramassive transfusion specifically, we have acknowledged the broader literature without adding references that might distract from the OLT focus of the manuscript.

Reviewer 1

We sincerely thank Reviewer 1 for the careful reading of our manuscript and for their insightful comments, which have substantially improved the clarity and interpretability of our work. We agree that each of the points raised is important and have addressed them in detail as follows.

4. Causality, intraoperative severity, and residual confounding

Reviewer's comment: "The main limitation of the study is that the analysis does not establish a causal relationship between transfusion volume and mortality. Patients receiving ultramassive transfusion are very likely those experiencing the most severe intraoperative complications (e.g., major bleeding, technically difficult surgery, graft issues), which are themselves strong predictors of mortality. Therefore, ultramassive transfusion is likely a marker of intraoperative severity rather than an independent cause of worse outcomes. Although propensity score matching was performed, the model appears to include mainly preoperative variables and donor characteristics, while important intraoperative severity markers such as blood loss, operative duration, vasopressor requirement, or intraoperative complications were not included. This creates a high risk of residual confounding and confounding by indication. This issue should ideally be addressed through additional clarification or analyses if possible, or at least more clearly acknowledged and discussed as a key limitation of the study."

Authors' response: We thank the Reviewer for raising this concern. We acknowledge that residual confounding and confounding by indication are inherent limitations of this retrospective propensity-matched study. Moreover, we agree that intraoperative variables, such as the volume of blood loss, surgical complexity and intraoperative complications, are likely important predictors of mortality. Unfortunately, this data was not captured in the transplant registry and was subsequently unavailable for our statistical analysis.

In response to this concern, we attempted to minimise confounding and to test the robustness of the primary findings. The propensity score matching strategy included 11 clinically relevant variables and achieved adequate covariate balance, with standardised mean differences (SMD) below 0.1 after matching (Figure 2; Supplementary Table 2). This balance post-matching demonstrates that the exposure groups in the primary analysis were well-matched on available characteristics. Moreover, this study was conducted at a single quaternary centre specialising in liver transplantation, ensuring internal consistency in clinical practice, which may mitigate residual confounding.

The consistency of the mortality signal across the unmatched and sensitivity analyses suggests that the UMT-mortality association may not be attributable to confounding alone. The association was observed in the unmatched cohort (n = 306), the primary matched cohort (n = 188), and the expanded cohort comparing UMT against all non-UMT (< 20 L intraoperative fluids) recipients (n = 376). Additionally, the mortality signal remained in the time-period sensitivity analysis to detect whether secular changes in clinical practice at our institution contributed to the UMT-mortality association.

Thus, this pattern of consistency suggests that the primary mortality findings are unlikely to be attributable to confounding alone, even in the absence of critical intraoperative variables.

To address this important concern, we have made the following changes:

I. **Strengthened limitations section:** We have expanded the Strengths and Limitations subsection to explicitly state that "critically, the propensity score model was constructed using preoperative variables only," and that key intraoperative factors influencing both transfusion volume and outcomes were unavailable. We now explicitly acknowledge confounding by indication, emphasizing that UMT "may reflect intraoperative severity rather than independently cause worse outcomes," and that the observed association "should be interpreted with caution."

II. **Clarified interpretation in Discussion and Conclusion:** We now consistently describe UMT as identifying a "high-risk" clinical phenotype and carefully avoid implying that UMT is itself causal. The Discussion explicitly notes that patients receiving UMT are plausibly those experiencing greater intraoperative complexity or catastrophic blood loss, and that this distinction cannot be resolved by our study design

III. **Survivor bias and unmeasured confounding:** We added a paragraph explicitly describing the possibility of survivor bias (patients must survive long enough to receive extreme volumes) and the broader issue of unmeasured confounding, underscoring that propensity score matching cannot adjust for variables that are not measured.

We believe these revisions make the limitations around causality and residual confounding much more transparent and aligned with the reviewer's concerns.

5. Exposure definition: total intraoperative fluid volume vs blood product units

Reviewer's comment: "A second concern relates to the definition of the exposure variable. Massive and ultramassive transfusion are defined using total intraoperative fluid volume, including crystalloids and colloids. This differs from most definitions in the literature, which typically rely on the number of blood product units. Including non-blood fluids may reflect differences in anaesthetic fluid management strategies rather than transfusion burden alone, which complicates interpretation of the results. The rationale for this definition should be explained more clearly. If this approach cannot be further justified or explored analytically, its implications for interpretation should be explicitly acknowledged and discussed as a limitation."

Authors' response: We thank the Reviewer for raising this concern. This concern was also raised by Reviewer 2. Accordingly, our pRBC-centred sensitivity analysis addresses both Reviewer comments.

We have addressed this in three ways:

I. Expanded justification in Methods: In the "Patient population and definitions" section, we now provide a clearer, a priori rationale for the composite fluid definition. Specifically, we emphasise that:

- The definition was chosen to capture the "total resuscitation burden", as all fluid components contribute to haemodilution, acid–base disturbance, endothelial injury, and overall physiological stress in OLT.

- Our approach is consistent with prior work at our institution, which also used a total-fluid definition in an OLT UMT cohort.

- Although not universal, these thresholds (10–20 L vs ≥20 L) represent clinically meaningful checkpoints for resource utilisation, and are aligned with local practice triggers for escalation of perioperative vigilance.

II. **Explicit acknowledgment as a limitation:** In the Strengths and Limitations section, we now explicitly state that our composite fluid definition "limits direct comparison with studies using pRBC-only thresholds" and may partly reflect differences in anaesthetic fluid management strategies rather than transfusion burden alone. We further highlight that "our findings may reflect differences in fluid management strategy rather than transfusion burden, introducing uncertainty when interpreting the significance of our findings.

III. **Additional analytic exploration (pRBC-centred sensitivity analysis):** To address the reviewer's suggestion for more analytic exploration, we have added a dedicated pRBC-centred sensitivity analysis (Sensitivity Analysis II) using pRBC units to define MT and UMT (10–14 vs ≥15 units), with full details in the Methods, Results, and Supplementary Tables 9–11. Although this analysis was underpowered at conventional UMT thresholds, it provides a complementary perspective and supports the notion that total fluid volume may be a more complete index of resuscitative burden in our setting.

We believe these additions provide a clearer justification of our exposure definition and appropriately frame it as both a strength (comprehensive resuscitation measure) and a limitation (comparability with pRBC-based definitions).

6. Spelling, typographical errors, and internal referencing issues

Reviewer's comment: "There are several minor issues that should be addressed during revision. Some spelling and typographical errors are present in the manuscript and should be corrected. In addition, several internal references appear to be incorrect or unresolved (e.g., "Error! Reference source not found."), suggesting formatting problems in the document. These should be carefully checked and corrected before publication."

Authors' response: We appreciate the reviewer drawing attention to these issues, which have now been addressed systematically. Specifically:

- We performed a comprehensive proofread of the manuscript to correct spelling, grammar, and typographical errors, including those in tables and figure legends.

- All internal referencing errors (e.g. "Error! Reference source not found.") arising from word-processor cross-references have been identified and corrected so that all tables, figures, and supplementary materials are now referenced correctly and consistently.

- We also standardised terminology (e.g., "ultramassive transfusion," "massive transfusion," and abbreviations) throughout the text for readability and consistency.

Reviewer 2

We are very grateful to Reviewer 2 for their thoughtful and constructive review, as well as the positive assessment of the study's contribution and statistical approach. We agree that each of the major and minor points raised is important and have revised the manuscript accordingly.

7. Overall assessment of robustness and contribution.

Reviewer's comment: "The PSM adjusts for preoperative variables only. Critical intraoperative factors (surgical complexity, operative duration, and bleeding aetiology) remain unmeasured. UMT may therefore reflect intraoperative factors rather than independently drive mortality, a distinction the design cannot resolve. The attenuation of the EAD association after matching illustrates this mechanism directly.

Authors' response: We thank the reviewer for these encouraging comments regarding the robustness of the mortality signal, the quality of the propensity score matching, and the transparent handling of secondary outcomes. This comment overlaps with Reviewer 1. We agree that the study design cannot definitively answer whether UMT independently drives mortality or whether it may be a proxy for intraoperative severity. A more detailed response to confounding factors is provided in response to Reviewer 1 Question 1 (see above) and applies here as well.

We have ensured that the manuscript clearly reports:

I. The **excellent covariate balance** achieved by the 1:1 optimal matching strategy, supported by Love plots and detailed standardised mean differences in Supplementary Tables.

II. The **consistency** of the mortality association across unmatched analyses and multiple sensitivity analyses, including the expanded comparator (UMT vs all non-UMT recipients) and the pRBC-centred analysis.

III. The "**underpowered nature**" of certain secondary and exploratory endpoints (e.g., graft loss, rare thrombotic events), with cautious interpretation explicitly noted in both Results and Discussion.

We appreciate the reviewer's recognition of these aspects and have retained this transparent framing in the revised manuscript.

8. Confounding vs independent risk and causal language

Reviewer's comment: "Causal language in the abstract and conclusion ("independently associated," "linked to") should be replaced throughout with "associated with," and the conclusion should explicitly acknowledge that causal inference is not supported."

Authors' response: We agree entirely with the reviewer that our design does not support causal inference and that the language should clearly reflect an "associational", rather than causal, interpretation.

Accordingly, we have made the following changes:

I. **Abstract:**
  - Replaced any phrases implying causality (e.g. "*independently associated*," "*linked to*") with the more neutral "associated with."
  - Added an explicit statement in the Abstract's Conclusion that "our study does not permit causal inference and the observed association may reflect intraoperative severity rather than a direct effect of transfusion volume."

II. **Main text and Conclusion:**
  - Throughout the Introduction, Discussion, and Conclusion, we systematically replaced causal-sounding terminology with "associated with," "*identifies a high-risk phenotype*," or "*correlated with*," as appropriate.

  - The Conclusion now explicitly states that "*due to the limitations of our study, our findings cannot establish a causal relationship; UMT may reflect intraoperative severity rather than independently drive mortality*."

III. **Limitations:**
  - The Strengths and Limitations section now prominently highlights that intraoperative factors were not included in the propensity model and that residual confounding and confounding by indication are likely.

We believe these revisions fully address the reviewer's concern and provide a transparent, appropriately cautious interpretation of our findings.

9. Composite fluid definition and additional pRBC-centred sensitivity analysis

Reviewer's comment: "Combining crystalloids, colloids, and blood products into a single threshold conflates physiologically distinct entities. A sensitivity analysis using a pRBC-centred definition would strengthen cross-study comparability and help disentangle haemodilution from transfusion-related immunomodulation."

Authors' response: We appreciate this insightful suggestion and agree that an additional pRBC-centred analysis would enhance comparability with existing literature and help disentangle mechanisms.

In response, we implemented the following:

I. **New pRBC-centred sensitivity analysis (Sensitivity Analysis II):**

  - We defined an MT group as "10–14 units pRBC" and a UMT group as "≥15 units pRBC", noting that the conventional threshold of ≥20 units pRBC yielded too few cases for meaningful analysis.

  - We performed 1:1 nearest-neighbour propensity score matching with a caliper of 0.2 standard deviations and assessed covariate balance using standardised mean differences (Supplementary Table 9).

  - We analysed patient and graft survival and secondary outcomes in this matched cohort, with results presented in Supplementary Tables 10–11 and Supplementary Figure 5.

- Although no associations reached statistical significance, likely due to limited sample size - the "direction of effect" (trend towards worse survival in the higher pRBC group) was consistent with our primary analysis based on total fluid volume.

The results of the pRBC-centred sensitivity analysis are included in the new supplementary material: Supplementary Table 9, Supplementary Table 10, Supplementary Table 11 and Supplementary Figure 5.

II. Discussion of composite vs pRBC-only definitions:

- The Discussion and Strengths and Limitations sections now explicitly discuss how our composite total-fluid definition may capture "overall resuscitative burden" and haemodynamic/haemodilutional stress, whereas pRBC-based definitions may more selectively reflect transfusion-related immunomodulation.

- We highlight that the attenuation of the mortality signal in the pRBC-centred analysis is consistent with the possibility that "total fluid volume" (including crystalloids and colloids) is a more comprehensive marker of intraoperative physiological stress in OLT.

III. Implications for future research:

- We now suggest that future OLT transfusion studies report both total fluid volume and pRBC units where possible, to allow clearer cross-study comparisons and mechanistic insights.

We are grateful for this recommendation, which has substantially enriched the analytic depth and interpretative nuance of the manuscript.

10. Ninety-day mortality effect estimate and model non-convergence

Reviewer's comment: "The 90-day mortality difference (11.7% vs. 0%) is the most striking finding, yet no effect size is reported because the Cox model did not converge. A Firth-corrected logistic regression estimate or exact confidence interval should improve the manuscript."

Authors' response: We thank the reviewer for highlighting this gap and fully agree that an effect estimate is important despite the issue of complete separation.

In the revised manuscript, we have:

I. Applied Firth's penalised regression methods:

- We used Firth's penalised logistic regression and Firth's penalised Cox regression to estimate the effect of UMT on 90-day mortality in the matched cohort, where complete separation precluded standard model convergence.

- These analyses were conducted using the `coxphf` package in R, as now detailed in the Statistical Analyses Methods section.

II. Reported effect sizes and confidence intervals:

- For 90-day mortality in the matched cohort, Firth's logistic regression produced an odds ratio of 26.03 (95% CI 3.31–3359.69, p = 0.001), which is now reported in Table 2.

- Firth's Cox regression yielded a hazard ratio of 24.30 (95% CI 3.17–3119, p = 0.001), reported in Table 3.

- We explicitly acknowledge the very wide confidence intervals, reflecting sparse events, and interpret these estimates as supportive, but not definitive, evidence reinforcing the strong 90-day mortality signal.[1]

III. Clarified methods and limitations:

- The Statistical Analyses section now states that Firth's penalised models were used specifically where standard models did not converge due to complete separation, and that proportional hazards diagnostics were not applicable in this context.

- The limitations section notes that these estimates should be interpreted with caution given the small number of events and resultant imprecision.

We believe these additions address the reviewer's concern by providing informative effect estimates while maintaining an appropriately cautious interpretation.

11. Typographical error in Table 1 and time-period sensitivity analysis

Reviewer's comment: "Table 1 contains a likely typographical error ("27.4.0 L"). A time-period sensitivity analysis would help assess whether secular changes in practice over the 15-year study window confound the association."

Authors' response: We appreciate this careful reading and agree that both issues are important.

I. Correction of typographical error:

- The typographical error in Table 1 has been corrected; the median total intraoperative fluid volume is now correctly reported as "27.4 L" (without the extraneous decimal point and zero).

- We also re-checked all numeric entries in Table 1 and the Supplementary Tables for similar formatting issues and corrected minor inconsistencies.

II. Time-period (era) sensitivity analysis (Sensitivity Analysis III)

- In line with the reviewer's excellent suggestion, we performed a "pre-specified era-based sensitivity analysis" to examine the potential impact of secular practice changes.

- We split the study period at the median transplant date (29 October 2018) into an "early era" (December 2009–October 2018) and a "contemporary era" (October 2018–December 2023).

- Rather than breaking matched pairs, we incorporated "transplant era" as a covariate in conditional Cox models for mortality, and additionally evaluated an interaction term (UMT × era).

- Era-adjusted conditional Cox models demonstrated that the UMT–mortality association persisted with hazard ratios virtually unchanged from the primary analysis (e.g., HR 2.55 [95% CI 1.06–6.11] for 3-year mortality and HR 2.54 [95% CI 1.17–5.49] for overall mortality), with no significant interaction between era and UMT status.

- These results are now presented and described in the Statistical Analyses and Sensitivity Analyses sections and summarised in Supplementary Table 12.

This analysis supports the view that secular changes in practice over the 15-year window are unlikely to fully explain the observed association between UMT and mortality.

Once again, our most sincere thanks to the Editor and the expert reviewers for dedicating their valuable time and expertise to review our manuscript. We deeply appreciate your thoughtful feedback and constructive suggestions, which have greatly contributed to improving the clarity and quality of our work. Your guidance has been instrumental in helping us refine our review, and we are grateful for the opportunity to benefit from your insights.

We hope that our manuscript is now suitable for publication in PLoS One. We look forward to hearing from you.

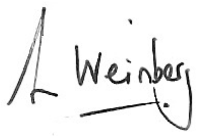

Professor Laurence Weinberg
BSc, MBBCh, MRCP, DipCritCareEcho, FANZCA, MD, PhD, ExecDipOL(Oxford)

Director, Department of Anaesthesia, Austin Hospital
Professor, Department of Critical Care, University of Melbourne, Australia
